# Comparative analysis of the complete chloroplast genomes of *Cirsium japonicum* from China and Korea

**DOI:** 10.1080/23802359.2021.1912669

**Published:** 2021-04-24

**Authors:** Lixia Tian, Mei Jiang, Haimei Chen, Jinglin Li, Linfang Huang, Chang Liu

**Affiliations:** Key Research Laboratory of Traditional Chinese Medicine Resources Protection, Administration of Traditional Chinese Medicine, National administration of Traditional Chinese Medicine, Institute of Medicinal Plant Development, Chinese Academy of Medical Sciences & Peking Union Medical College, Beijing, China

**Keywords:** *Cirsium japonicum*, chloroplast genome, identification, phylogenetic analysis

## Abstract

*Cirsium japonicum* (*C. japonicum*) is a traditional Chinese medicine belonging to the family Asteraceae. The previous studies have indicated that the chemical compound content of *C. japonicum* from different places was different. To distinguish *C. japonicum* from different geographies, the chloroplast genome of *C. japonicum* from China was sequenced and compared with that from Korea. The total length of this genome is 152,602 bp, similar to that of Korea (152,606 bp). It has a conservative quartile structure which is composed of a large single-copy (LSC) region, a small single-copy (SSC) region and a pair of inverted repeats (IRs) regions, with lengths of 83,487 bp, 18,721 bp, and 25,197 bp, respectively. It encodes 79 protein-coding, 27 transfer RNAs, and 4 ribosomal RNA genes. The overall GC content of the genome is 37.70%. A total of 20 single nucleotide polymorphisms and 6 insertions and deletions were identified between the chloroplast genome of *C. japonicum* from China and Korea. These results can be applied to develop molecular markers to distinguish *C. japonicum* from different geographical origins.

*C. japonicum*, belonging to *Cirsium* genus, is a perennial herb distributed in China, as well as Japan and Korea, which has a long history of being used as functional food and herb (Zeng et al. 2016). Previous reports have shown that it has multiple pharmacologic effects, including antidiabetic, antitumor, and anti-inflammatory (Ma et al. 2016; Yang et al. 2018; Zhao et al. 2018; Jang et al. 2020). The chemical compound content of *C. japonicum* from different places is different, which may affect clinical efficacy (Chen and Gong 2013). The chloroplast genome is a perfect option for developing molecular markers to discriminate the different geographical areas of the same species (Jiang et al. 2018). Hence, the chloroplast genome of *C. japonicum* from china was sequenced and compared with that from Korea to find the potential maker.

The fresh and healthy leaves of the *C. japonicum* were harvested from the Central china medicinal botanical garden, Hubei, China (30°10′42″N, 109°44′55″E). The specimen was stored under voucher number Implad201808173 at the Herbarium of Institute of Medicinal Plant Development in Beijing, China. DNA was extracted followed the modified CTAB method. Subsequently, the integrity and concentration of extracted DNA were measured by electrophoresis in 1% (w/v) agarose gel and spectrophotometer (Nanodrop 2000, Thermo Fisher Scientific, Waltham, MA). The DNA library with insert sizes of 500 bases was constructed with 1 ug DNA using the library preparation kit (New England BioLabs, Ipswich, MA), then was subjected to high-throughput sequencing using an Illumina Hiseq2000 sequencer (Illumina Inc., San Diego, CA). Clean reads were assembled using NOVOPlasty v.2.7.2 (Dierckxsens et al. 2017). The assembled chloroplast genome was annotated with CPGAVAS2 (Shi et al. 2019) and then edited using Apollo (Misra and Harris 2006). The genome sequence and annotations have been deposited in GenBank with accession numbers MW035606.

The chloroplast genome of *C. japonicum* from China was 152,602 bp, similar to that from Korea (152,606 bp), comprising of a large single-copy (LSC) region of 83,487 bp, a small-copy (SSC) region of 18,721 bp regions, and a pair of inverted repeats (IRs) regions of 25,197 bp. The GC content of the total genome, LSC, SSC, and IR regions is 37.70%, 35.85%, 31.38%, and 43.10%, respectively. Besides, the genome encodes a total of 110 genes, including 79 protein-coding genes, 27 tRNA genes and 4 rRNA genes, respectively. Overall, the genome is highly consistent with the one previously reported.

To detect the variation between the chloroplast genome of *C. japonicum* from China and Korea, the DnaSP v6.0 (Rozas et al. 2017) software was employed. A total of 20 single nucleotide polymorphisms (SNPs) and 6 insertions and deletions (InDels) were detected. Most SNPs were detected in the LSC and SSC regions, with 10 and 6, respectively, and 4 were found in the IR regions. Among the protein-coding genes, the SNPs were found in *ycf*1, *rps*16, and *trnE*-UUC genes, resulting in the changes of three amino acids (836nd, Ser-Arg; 1239nd, His-Asn; 1342nd, Leu-termination codon), one (259nd, Ser-Leu) and one (89nd, Arg-Lys), respectively. The InDels were found in the *trn*K-UUU and *mat*K genes. Our results showed that 5 protein-coding genes (*ycf*1, *rps*16, *trn*E-UUC, *trn*K-UUU, and *mat*K) might be effective in distinguishing *C. japonicum* from China and Korea.

Finally, we inferred phylogenetic relationships among 6 *Cirsium* species based on the complete chloroplast genome sequences. Two Cynara species were used as outgroups. The chloroplast genome sequences of 6 species were downloaded from GenBank (the accession numbers are shown in Figure 1). Shared protein sequences were aligned using the CLUSTALW2 (v2.0.12) program. The phylogenetic tree was constructed using the maximum-likelihood (ML) method implemented in RaxML (v8.2.4) (Stamatakis 2014). The bootstrap analysis was performed with 1000 replicates. In the phylogenetic tree, most nodes have high bootstrap support values that are >80%. Figure 1 shows that the two *C. japonicum* species are clustered together, as expected. And they are closely related to *Cirsium rhinoceros*. The present study indicated that the chloroplast genome can be used to identify *C. japonicum* from different origins .

**Figure 1.**
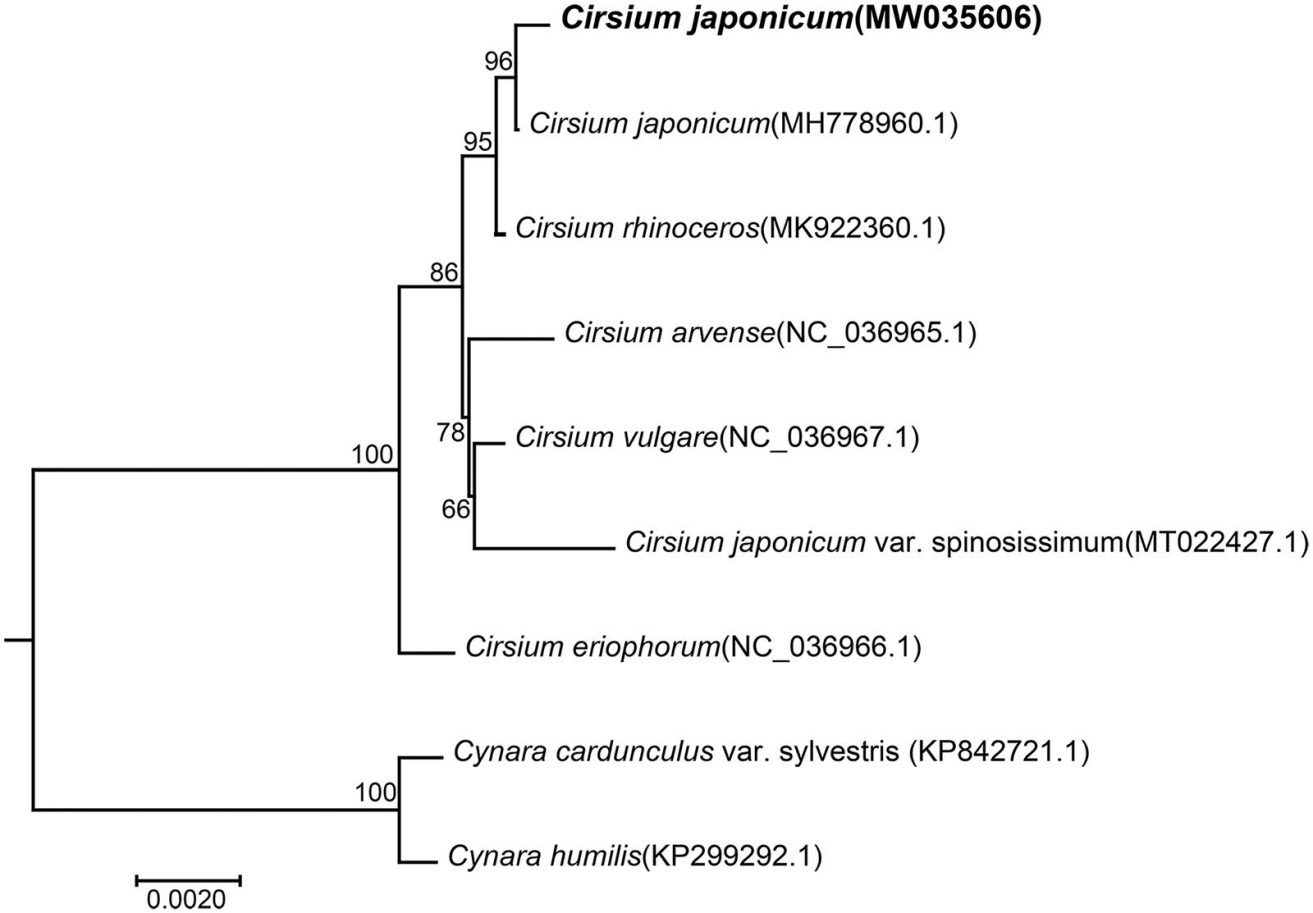
Phylogenetic relationships of *Cirsium* species inferred using maximum-likelihood (ML) method. The phylogenetic tree constructed using the shared protein sequences among the 9 samples. The number on the branch indicates the bootstrap value. *Cynara humilis* (KP299292.1) and *Cynara cardunculus* var. sylvestris (KP842721.1) were used as outgroups.

## Data Availability

The data that support the findings of this study are openly available in GenBank at https://www.ncbi.nlm.nih.gov/nuccore/MW035606. Raw sequencing data used in this study have been deposited in SRA with accession number SRR12975765.
